# Anatomical and functional outcomes of retinal detachment associated with nontraumatic giant retinal tears compared to simple rhegmatogenous retinal detachment

**DOI:** 10.1186/s40942-022-00407-y

**Published:** 2022-09-15

**Authors:** Jérôme Garneau, Mélanie Hébert, Eunice You, Serge Bourgault, Mathieu Caissie, Éric Tourville, Ali Dirani

**Affiliations:** 1 Faculty of Medicine, Université Laval, Quebec, Canada; 2 Department of Ophthalmology, Centre Universitaire d’Ophtalmologie, CHU de Québec - Université Laval (Hôpital du Saint-Sacrement), 1050 Chemin Ste Foy, Quebec, QC G1S4L8 Canada

**Keywords:** Giant retinal tear, Retinal detachment, Pars plana vitrectomy, Scleral buckle

## Abstract

**Background:**

To compare the functional and anatomical outcomes of primary surgery in patients with giant retinal tear (GRT)-associated retinal detachment (GRT-RD) to patients with simple rhegmatogenous RD (RRD).

**Methods:**

This is a retrospective study at the CHU de Québec - Université Laval. Medical records of all consecutive patients operated for RD between 2014 and 2018 were reviewed. Patients with GRT-RD and RRD were included. Preoperative, intraoperative, and postoperative data were compared between both groups, including extension of giant tears, number of RD quadrants, preoperative macula and lens status, type of surgery, best corrected visual acuity (BCVA) in logarithm of the minimum angle of resolution (logMAR) preoperatively and at follow-up, and single surgery anatomical success (SASS).

**Results:**

There were 39 patients (1.7%) with GRT-RD and 1661 patients (74%) with RRD. Median [Q1, Q3] ages were 59 [52, 62] years and 62 [56, 69] years (p = 0.003), while number of affected quadrants were 2 [2, 3] and 2 [2, 3] (p = 0.96) in GRT-RD and RRD patients, respectively. In GRT-RD patients, GRT size was 120 [90, 150] degrees. Final BCVA was 0.30 [0.10, 0.30] and 0.30 [0.10, 0.40] (p = 0.76) in GRT and RRD patients, respectively. SSAS was 82% (32/39) in the GRT-associated-RD group and 90% (1495/1661) in the RRD group (p = 0.10). After correcting for other preoperative factors, GRT was a risk factor for worse SSAS (odds ratio: 0.422, p = 0.047).

**Conclusions:**

GRT-RD is still challenging to treat, and our results suggest that it is a risk factor for poorer SSAS.

## Background

Giant retinal tears (GRTs) are defined as full thickness neurosensory retinal breaks extending circumferentially for 90° (3 clock hours) or more in the presence of posterior vitreous detachment (PVD) [1]. Different risk factors have been identified for GRT such as high myopia, trauma, pseudophakia and aphakia, and connective tissue diseases (e.g., Marfan syndrome, Stickler syndrome). The majority of GRTs are idiopathic and occur spontaneously [2].

GRTs can lead to the development of retinal detachment (RD), causing rapid and important loss of vision in most patients. It is estimated that GRTs are responsible for 0.5 to 8.3% of all RD [3–6]. In such cases, surgery is required to reattach the retina and optimize final functional outcomes. Like in simple rhegmatogenous RD (RRD), both pars plana vitrectomy (PPV) and PPV with scleral buckle (SB) (PPV-SB) are commonly used for management of GRT-associated-RD (GRT-RD). However, an increased tendency for proliferative vitreoretinopathy (PVR) development and greater technical requirements contribute to poorer outcomes in these patients [7, 8]. GRT-RD treatment remains a challenge for retinal surgeons and anatomical success of primary surgery tends to be lower than for RRD.

Few studies from a single center directly compared the qualitative and quantitative characteristics between GRT-associated-RD and simple RRD patients. The aim of this study is to analyze the anatomical and functional outcomes in GRT-RD and simple RRD patients, including rate of primary surgery success and final visual acuity. The secondary objective is to identify any factors predictive of a successful surgical outcome and better visual outcome.

## Methods

**Fig. 1:**
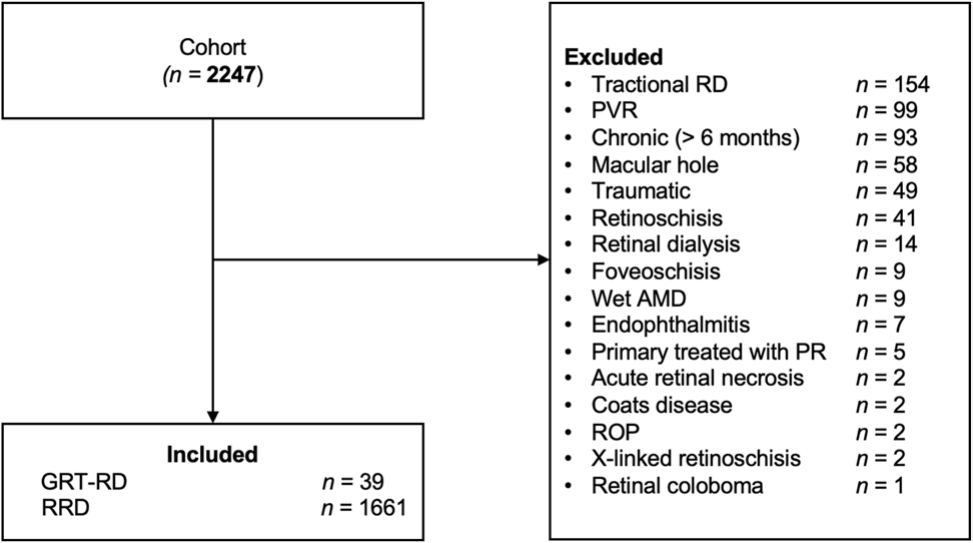
Patient selection flow chart in the cohort of retinal detachment (RD) patients. *AMD* age-related macular degeneration, *PR* pneumatic retinopexy, *PVR* proliferative vitreoretinopathy, *ROP* retinopathy of prematurity

This is a retrospective study of all patients operated for GRT-RD (n = 39) or RRD (n = 1661) at the Centre hospitalier universitaire de Québec - Université Laval between 2014 and 2018. It adhered to the tenets of Declaration of Helsinki and the Institutional Review Board of the CHU de Québec (Reference number 2020:4798). Individual patient consent was waived given the retrospective nature of the study. All patients were operated for RD by one of five fellowship certified attending vitreoretinal surgeons. Surgery choice and tamponade agent used were at the discretion of the treating surgeon and were comparable between all surgeons. Patients with secondary causes of RD, including traumatic RD (including traumatic GRT), tractional diabetic RD, RD with preoperative PVR ≥ C2, retinoschisis, retinal dialysis, and macular holes were excluded. Patients treated with pneumatic retinopexy as primary surgery were also excluded (shown in Fig. 1). All ophthalmology follow-ups until March 2020 were reviewed and included for analysis. Patients needed to have a minimum follow-up of 3 months to be included.

We collected complete preoperative, intraoperative, and postoperative data for all included patients from baseline evaluations including visual acuity, ophthalmic history, complete slit lamp exam, and dilated fundus examination, intraoperative surgeon observations, and follow-up visits. Preoperative data included age, sex, laterality, best-corrected visual acuity (BCVA) at baseline, lens status (i.e., phakic, pseudophakic, or aphakic), macula status (i.e., on, off, or split), myopia ≥ 4 diopters, the number of RD quadrants, the size of the giant retinal tear for the GRT group, and the duration of symptoms before first surgery. Macula status was determined through clinical examination, but in cases which were unclear, optical coherence tomography (OCT) were reviewed to confirm macula status. RD not involving the fovea were defined as “macula on”, RD fully involving the fovea were considered “macula off”, and RD partially involving the fovea were considered “macula split.”

The presence of a GRT or an RD was evaluated based on clinical assessment by a surgical retina specialist. Classification of patients as having GRT-RD or RRD, as well as classification of the size of GRT are based on the preoperative and intraoperative assessment of the RD. All retinal specialist used the standard definition of GRT (i.e., 90° or more) to make the diagnosis. In this study, we defined all full-thickness retinal defects as retinal breaks. The number of retinal breaks was noted in preoperative evaluations and confirmed by direct observation intraoperatively. The presence of PVR was also confirmed by notation on surgery protocol. Operative data included the type of surgery and tamponade agent used for the primary surgery. Postoperative data included BCVA at 3 months and at the final follow-up, the development of visually significant cataracts requiring surgery, and the total duration of follow-up. Primary outcome was single surgery anatomical success (SSAS) defined as the absence of RD recurrence requiring an additional surgical intervention at any time during follow-up. Laser procedures performed in the outpatient clinic were not considered reoperations and patients needed to be followed for a minimum of 3 months to confirm SSAS.

Surgical data were further analyzed to determine the influence of initial surgery choice on SSAS. To compare the surgical anatomical and functional success, we focused on the two main surgeries performed for the treatment of RD: PPV alone and PPV-SB. We also compared the SSAS for patients with GRT-RD against patients with RRD.

### Statistical analysis

Data are presented as mean ± standard deviation for continuous, normally distributed variables, as median [first quartile, third quartile] for continuous, non-normally distributed variables, and as frequencies (percentages) for categorical variables. Characteristics and variables were compared between the two groups (i.e., PPV and PPV-SB) using independent Student’s t-test or Mann–Whitney U test for continuous variables and Chi-square analysis or Fisher’s exact test for categorical variables, as appropriate. Shapiro–Wilk test and Q–Q plots with 95% confidence intervals were used to test for normal distribution of continuous variables.

Vision was converted from metric Snellen notation to logarithm of the minimum angle of resolution (logMAR) for analysis purpose [9]. Vision levels of counting fingers, hand motion, light perception, and no light perception were assigned Snellen visual acuity values of 1.0/200, 0.5/200, 0.25/200, and 0.125/200 (logMAR equivalent 2.3, 2.6, 2.9, and 3.2, respectively) [10, 11].

A multiple logistic regression model was built for SSAS. All relevant preoperative and intraoperative characteristics were considered for inclusion. These include age, sex, pseudophakia, myopia greater than 4 diopters, presence of GRT, baseline VA, duration of symptoms at presentation, RD quadrants involved, preoperative macula status, type of surgery, type of gas tamponade. Variables were included in the model while respecting a minimum of 5–9 events per predictor variable [12]. A backwards elimination strategy was used to manually select variables for the final model, with variables p > 0.2 removed. Odds ratios (OR) with 95% confidence intervals (CI) were produced for each variable included. Number of RD quadrants, macula status, and visual acuity at presentation were not added in the final model because of collinearity with the presence of inferior tears in inferior RD variable which was more closely associated with the outcome. Aphakia was also not considered given that no patient in the GRT group was aphakic and only 11 patients in the RRD group were aphakic.

Statistical analyses were performed using R for Windows (version 3.6.3; R Foundation for Statistical Computing) and IBM SPSS Statistics for Windows (version 27.0; IBM Corp., Armonk, NY). Analyses were conducted at the 0.05 significance level.

## Results

### Patient baseline characteristics

During the study period, among 2247 patients operated for RD, 39 eyes (1.7%) were operated for GRT-RD and 1661 eyes (74%) for simple RRD. Baseline characteristics by group are presented in Table 1. Median [Q1, Q3] age at the time of primary surgery was significantly greater among the RRD group compared to the GRT-RD group (62 [56, 69] years vs. 59 [52, 62] years; p = 0.003). There was no significant difference in macular status at presentation between the GRT-RD group compared to the RRD group preoperatively (p = 0.07). There were no other differences among baseline characteristics, including number of RD quadrants, baseline BCVA, or duration of symptoms. In the GRT-RD group, the median size of the GRT at baseline was 120 [90, 150] degrees. In RRD patients, the median [Q1, Q3] number of retinal tears within the retinal detachment was 2 [1, 3].

**Table 1:** Preoperative baseline characteristics, initial operative management, and visual outcomes at follow-up of 39 patients with giant retinal tear-associated retinal detachments (GRT-RD) and 1661 patients with simple rhegmatogenous retinal detachments (RRD)

Characteristic, n (%) or median [Q1, Q3]	GRT-RD n = 39	RRD n = 1661	p-value
Preoperative characteristics			
Age, years	59.0 [52.0, 62.0]	62.0 [56.0, 69.0]	0.003
Male sex	29 (74%)	1049 (63%)	0.15
Duration of symptoms, days	7 [5, 13]	7 [4, 15]	0.73
Asymptomatic patients	0 (0%)	21 (1%)	1.00
Baseline visual acuity, logMAR	0.30 [0.10, 2.30]	0.54 [0.10, 2.30]	0.14
Snellen	20/40	20/70	
Affected eye (left)	21 (54%)	805 (49%)	0.51
Myopia, > 4 diopters	7 (18%)	352/1620 (22%)	0.57
Lens status			0.30
Aphakic	0 (0%)	11 (0.7%)	
Phakic	26 (67%)	907 (55%)	
Pseudophakic	13 (33%)	743 (45%)	
Macula status			0.07
On	21 (54%)	595 (36%)	
Off	15 (38%)	884 (53%)	
Split	3 (8%)	182 (11%)	
Number of quadrants affected	2.0 [2.0, 3.0]	2.0 [2.0, 3.0]	0.96
Intraoperative management			
Primary surgery type			0.07
SB, n (%)	0 (0%)	113 (7%)	
PPV, n (%)	16 (41%)	827 (50%)	
PPV-SB, n (%)	23 (59%)	721 (43%)	
Primary surgery tamponade agent			< 0.001
None, n (%)	0 (0%)	30 (2%)	
Air, n (%)	0 (0%)	9 (1%)	
SF_6_, n (%)	10 (26%)	1311 (79%)	
C_3_F_8_, n (%)	28 (72%)	291 (18%)	
Silicone oil, n (%)	1 (3%)	20 (1%)	
Postoperative outcomes			
Recurrence of retinal detachment	7 (18%)	166 (10%)	0.10
Visual acuity at 3 months, logMAR	0.30 [0.18, 0.65]	0.30 [0.10, 0.54]	0.13
Snellen	20/40	20/40	
Final visual acuity, logMAR	0.30 [0.10, 0.30]	0.18 [0.10, 0.40]	0.91
Snellen	20/40	20/30	
Follow-up time, months	17.0 [4.0, 28.0]	11.0 [3.0, 25.0]	0.05
Cataract development*	19/26 (73%)	687/907 (76%)	0.75

^*^Cataract development was calculated over the number of phakic patients preoperatively who could develop a cataract postoperatively

### Intraoperative management

The primary surgery type performed and choice of tamponade agent by group are presented in Table 1. Principal surgery performed in the entire cohort was PPV (n = 843, 50%) and PPV-SB (n = 744, 43%) and there was no statistically significant difference between the GRT-RD and the RRD groups regarding procedure choice (p = 0.07). None of the patients in the GRT-RD group were treated with SB alone, and 7% of RRD group had SB alone. A significantly greater number of patients in the GRT-RD group received C_3_F_8_ as primary tamponade agent compared to the RRD group (n = 28, 72% vs. n = 291, 18%) (p < 0.001).

### Postoperative outcomes

The median total duration of follow-up was 17 [4, 28] months and 11 [3, 25] months in the GRT-RD and RRD groups, respectively (p = 0.05). At 3 months and at final follow-up, BCVA was not significantly different between RRD patients compared to GRT-RD patients.

SSAS was achieved in 32 (82%) GRT-RD patients, while SSAS was achieved in 1495 (90%) RRD patients (p = 0.10). PVR was the cause of surgery failure for all 7 patients who had RD recurrences in the GRT-RD group and among them, two patients underwent PPV alone as primary surgery (n = 2/16, 13%) and five patients underwent PPV-SB (n = 5/23, 22%) (p = 0.68). For the RRD patients, the success rate of PPV alone and PPV-SB was also similar. Among those with RD recurrences, 76 patients were treated with PPV alone (76/827, 9%) and 80 patients were treated with PPV-SB (80/721, 11%) (p = 0.21). Among patients who had recurrences in the RRD group, 133 (8%) required a second surgery, while 31 (2%) required three and 2 (0.1%) required four surgeries. This was similar to patients in the GRT-RD group who required two surgeries in 4 patients (10%), three surgeries in 2 (5%) and four surgeries in 1 (2.6%) (p = 0.09). Final retina status was “on” for 39 (100%) of GRT-RD patients compared to 1655 (99.6%) of RRD patients (p = 1.00).

### Multiple regression analysis for SSAS

The final multiple logistic regression model for SSAS is presented in Table 2. The variables included in the model were: age, sex, duration of symptoms, presence of myopia, presence of GRT, macula status and pseudophakia. Number of RD quadrants, and visual acuity at presentation were not added in the final model because of collinearity. Aphakia was also not considered given that no patient in the GRT group was aphakic and only 11 patients in the RRD group were aphakic. After correction for other preoperative factors, GRT was associated with a significantly decreased rate of SSAS (OR 0.422, 95% CI 0.190–1.071; p = 0.047).

**Table 2:** Multiple logistic regression model for associations between clinical characteristics and single surgery anatomic success (SSAS) rate following retinal detachment (RD) repair in giant retinal tear (GRT) associated RD (n = 39) and simple rhegmatogenous RD (n = 1661)

Characteristic	OR (95% CI); p-value
Age, years	0.988 (0.971, 1.003); 0.14
Male sex	0.880 (0.610, 1.255); 0.48
GRT	0.422 (0.190, 1.071); 0.047
Duration of symptoms	1.015 (1.003, 1.027); 0.02
Myopia, > 4 diopters	0.793 (0.524, 1.223); 0.28
Macula status	
On	REF
Off	0.970 (0.668, 1.399); 0.87
Split	1.922 (0.974, 4.245); 0.08
Pseudophakia	1.314 (0.905, 1.919); 0.15

## Discussion

In this study, we compared the anatomical and functional outcomes of 39 eyes with GRT-RD to 1661 eyes with simple RRD. The prevalence of GRT-RD of 1.7% in our cohort is comparable with previous studies reporting a prevalence of 0.5 to 8.3% [3–6]. A Scottish study reported a GRT-RD prevalence of 1.3% among 1130 patients with RD [13].

The baseline characteristics between both groups differed significantly for age. In our study, GRT-RD patients were significantly younger than RRD patients at presentation. It is known that giant retinal tears affect younger patients, with a mean age of 30 to 53 years old in previous reports [14–21]. While the median age of GRT-RD patients in our study is greater than that reported in the literature, this discrepancy can be explained by the fact that we did not include traumatic GRT. Trauma is an important risk factor for developing a GRT and younger patients are more susceptible to experiencing a trauma. There is also a known preponderance of GRT in men than in women [2]. In our study, 29 patients (74%) in the GRT-RD group were men, which is consistent with a male prevalence between 65 and 91% previously reported [22]. Meanwhile, there was no significant difference between the BCVA and the macular status of patients with GRT-RD or RRD at baseline. The median BCVA at presentation was 20/40 for GRT-RD group, which is within the expected range observed in GRT [23]. While traumatic GRT and PVR grade C2 or greater may be expected to have a higher prevalence of detached macula, these cases had been excluded.

The BCVA at 3 months of follow-up and at final follow-up were similar between both groups. The high proportion of patients being macula “on’’ at baseline in the GRT-RD group and the fact that traumatic GRT were excluded could have contributed to better visual outcomes.

In our study, the primary reattachment rate was not significantly different between GRT-RD and RRD patients (82% vs 90%, p = 0.10). Previously published studies have stated a primary anatomical success rate between 68 and 96% in GRT-RD patients, which is concordant with our results [22]. The British Giant Retinal Tear Epidemiology Eye Study (BGEES) prospectively analyzed 60 patients (62 eyes) with GRT-RD. They had a primary anatomical success rate of 87.7%, which is also compatible with our analysis [23]. Our multiple regression analysis however suggests that GRT might be associated with poorer anatomical outcomes in the primary repair of RD. The bigger size of the retinal tears and increased propensity for PVR development in GRT-RD patients may be plausible explanation. The final anatomical success rate in our study was 100% (39/39) and 99.6% (1655/1661), which is consistent with previous reports (81% to 100%) [22].

Different types of surgery exist for the repair of GRT-RD but PPV alone and PPV combined with a SB (PPV-SB) remain the most frequently performed. In our study, the primary anatomical success rate was not significantly different between PPV and PPV-SB in GRT-RD patients (88% vs. 78%; p = 0.68). In 2018, Rodriguez et al. analyzed 80 eyes treated with SB, PPV or PPV-SB for GRT-RD and found no differences in the primary anatomical success rate between the three groups [24]. Another study of 94 Indian patients with GRT-RD also stated no differences in the anatomical outcomes of primary surgery between PPV and PPV-SB [25]. The addition of a scleral buckle is particularly recommended if the edge of the GRT is not inverted and in cases of PVR [2]. In other circumstances, the role of scleral buckle is still controversial.

To our knowledge, our study is the only large population study that directly compares the anatomical and functional outcomes of retinal detachment associated with a giant retinal tear with simple RRD.

## Limitations

This is a retrospective, single center study. We describe 39 patients with GRT in this cohort, which was insufficient to build a multivariable model examining rates of SSAS within this group only. Given the small number of patients with GRT, the effect of GRT on SSAS yields a wide confidence interval encompassing the threshold of OR = 1, even though the p-value is deemed statistically significant (OR 0.422, 95% CI 0.190–1.071; p = 0.047). We also could not study specific interventions to improve success rates in GRT-RD including using multiple linear regression for final VA given the small sample size of GRT.

Outcome data was collected until final follow-up. Rates of outcomes such as cataract development and recurrence of detachment could however be affected by total length of follow-up, which was restricted to 3 months in several patients.

## Conclusion

In conclusion, our retrospective cohort study highlights that the initial presentation and clinical course of RD patients with GRT may differ from simple RRD patients. Management of GRT-RD also remains more challenging with potentially less favorable anatomical and functional outcomes compared to RRD. When adjusting for other preoperative risk factors, GRT remained significantly associated with reduced SSAS compared to simple RRD. Standard PPV and combined PPV-SB showed similar surgical outcomes in repairing the RD associated with a giant retinal tear. More studies are needed to optimize surgical outcomes in GRT-RD.

## Data Availability

The datasets used and/or analyzed during the current study are available from the corresponding author on reasonable request.

## Abbreviations

BCVA: Best-corrected visual acuity
C_3_F_8_: Perfluoropropane
CI: Confidence intervals
GRT: Giant retinal tear
GRT-RD: Giant retinal tear-associated retinal detachment
logMAR: Logarithm of the minimum angle of resolution
OCT: Optical coherence tomography
OR: Odds ratio
PPV: Pars plana vitrectomy
PPV-SB: Pars plana vitrectomy with scleral buckle
PVD: Posterior vitreous detachment
PVR: Proliferative vitreoretinopathy
RD: Retinal detachment
RRD: Rhegmatogenous retinal detachment
SB: Scleral buckle
SD: Standard deviation
SF6: Sulfur hexafluoride
SSAS: Single surgery anatomical success
VA: Visual acuity
